# Weakly Supervised Deep Learning Predicts Immunotherapy Response in Solid Tumors Based on PD-L1 Expression

**DOI:** 10.1158/2767-9764.CRC-23-0287

**Published:** 2024-01-11

**Authors:** Marta Ligero, Garazi Serna, Omar S.M. El Nahhas, Irene Sansano, Siarhei Mauchanski, Cristina Viaplana, Julien Calderaro, Rodrigo A. Toledo, Rodrigo Dienstmann, Rami S. Vanguri, Jennifer L. Sauter, Francisco Sanchez-Vega, Sohrab P. Shah, Santiago Ramón y Cajal, Elena Garralda, Paolo Nuciforo, Raquel Perez-Lopez, Jakob Nikolas Kather

**Affiliations:** 1 Radiomics Group, Vall d'Hebron Institute of Oncology (VHIO), Barcelona, Spain.; 2 Molecular Oncology Group, Vall d'Hebron Institute of Oncology (VHIO), Barcelona, Spain.; 3 Else Kroener Fresenius Center for Digital Health, Technical University Dresden, Dresden, Germany.; 4 Pathology Department, Vall d'Hebron University Hospital (VHUH), Barcelona, Spain.; 5 Oncology Data Science (ODysSey) Group, Vall d'Hebron Institute of Oncology (VHIO), Barcelona, Spain.; 6 Assistance Publique-Hôpitaux de Paris, Département de Pathologie, CHU Henri Mondor, Créteil, France.; 7 Université Paris-Est Créteil, Faculté de Médecine, Créteil, France.; 8 Biomakers and Clonal Dynamics Group, Vall d'Hebron Institute of Oncology (VHIO), Barcelona, Spain.; 9 Department of Pathology and Laboratory Medicine, Children's Hospital of Philadelphia, Philadelphia, Pennsylvania.; 10 Department of Pathology and Laboratory Medicine, Memorial Sloan Kettering Cancer Center, New York, New York.; 11 Computational Oncology, Memorial Sloan Kettering Cancer Center, New York, New York.; 12 Department of Medical Oncology, Vall d'Hebron University Hospital and Institute of Oncology (VHIO), Barcelona, Spain.; 13 Pathology and Data Analytics, Leeds Institute of Medical Research at St James's, University of Leeds, Leeds, United Kingdom.; 14 Department of Medicine I, University Hospital Dresden, Dresden, Germany.; 15 Medical Oncology, National Center for Tumor Diseases (NCT), University Hospital Heidelberg, Heidelberg, Germany.

## Abstract

Programmed death-ligand 1 (PD-L1) IHC is the most commonly used biomarker for immunotherapy response. However, quantification of PD-L1 status in pathology slides is challenging. Neither manual quantification nor a computer-based mimicking of manual readouts is perfectly reproducible, and the predictive performance of both approaches regarding immunotherapy response is limited. In this study, we developed a deep learning (DL) method to predict PD-L1 status directly from raw IHC image data, without explicit intermediary steps such as cell detection or pigment quantification. We trained the weakly supervised model on PD-L1–stained slides from the non–small cell lung cancer (NSCLC)-Memorial Sloan Kettering (MSK) cohort (*N* = 233) and validated it on the pan-cancer-Vall d'Hebron Institute of Oncology (VHIO) cohort (*N* = 108). We also investigated the performance of the model to predict response to immune checkpoint inhibitors (ICI) in terms of progression-free survival. In the pan-cancer-VHIO cohort, the performance was compared with tumor proportion score (TPS) and combined positive score (CPS). The DL model showed good performance in predicting PD-L1 expression (TPS ≥ 1%) in both NSCLC-MSK and pan-cancer-VHIO cohort (AUC 0.88 ± 0.06 and 0.80 ± 0.03, respectively). The predicted PD-L1 status showed an improved association with response to ICIs [HR: 1.5 (95% confidence interval: 1–2.3), *P* = 0.049] compared with TPS [HR: 1.4 (0.96–2.2), *P* = 0.082] and CPS [HR: 1.2 (0.79–1.9), *P* = 0.386]. Notably, our explainability analysis showed that the model does not just look at the amount of brown pigment in the IHC slides, but also considers morphologic factors such as lymphocyte conglomerates. Overall, end-to-end weakly supervised DL shows potential for improving patient stratification for cancer immunotherapy by analyzing PD-L1 IHC, holistically integrating morphology and PD-L1 staining intensity.

**Significance:**

The weakly supervised DL model to predict PD-L1 status from raw IHC data, integrating tumor staining intensity and morphology, enables enhanced patient stratification in cancer immunotherapy compared with traditional pathologist assessment.

## Introduction

Over the last decade, tumor immunotherapy has irrevocably altered the landscape of oncology. Checkpoint inhibitors, in particular, are now employed in practically all tumor types (1–3). These treatments are increasingly being employed in early stages, palliative lines of therapy in advanced cancer, as well as adjuvant or neoadjuvant therapy. However, the identification of suitable patients remains a critical issue, as only a subset of individuals reacts to checkpoint inhibition. The immunotherapy biomarker which is most commonly used for patient selection in clinical practice is the programmed death-ligand 1 (PD-L1) status, which is assessed using IHC assays (4–6). Pathologists typically assess these stains by visual inspection of the sample. The number of positive tumor cells and positive immune cells are visually estimated. Nevertheless, manual estimation of a biomarker is obviously not desirable for such a decisive therapy recommendation, and multiple studies have demonstrated that pathologists exhibit a considerable level of variability in this assessment (7–10). Hence, a more precise and reliable approach is imperative to ensure accurate diagnosis and treatment.

As a result, dozens of studies have been conducted to build image analysis tools that assess the PD-L1 status of a tissue sample (11). The majority of these systems mimic the human workflow explicitly: First, they are recognizing tissue on a slide. Second, all individual cells are recognized. These cells are classified into relevant categories, such as tumor cells or immune cells. Finally, the color intensity of each individual cell is evaluated, and the cell is classified as positive or negative (12–16). Although this procedure is theoretically relatively well defined, there are issues with the approach of such a multistep pipeline in practice. If the tissue slice differs from the slices in the training dataset, for example due to artifacts or a difference in sample handling, and even if only one of the intermediary steps of the pipeline is confused here, the final result is untrustworthy. Consequently, another study used deep learning (DL) to combine PanCytokeratin and PD-L1–stained images to differentiate tumor regions from epithelial tissue and assess the proportion of PD-L1–positive inside the tumor areas as a multilabel task (17). Other studies investigated as an alternative the implementation of DL methods to predict PD-L1 status from other image types such as hematoxylin and eosin (H&E) histology images (18, 19) or from radiology images as a surrogate for immunotherapy response (20).

An alternative to such multistep image analysis pipelines is a weakly supervised end-to-end approach. In such an approach, a DL network is trained to predict a biomarker directly from the digitized whole-slide images (WSI) without intermediate steps such as the detection of individual cells being explicitly modeled (21–23). Through the implementation of attention-based methods, the DL model is trained to identify distinct patterns across the different regions of the sample that are contributing to the PD-L1 status. As a result, the model can learn to focus on the areas containing tumor cells and avoiding nonspecific tissue from the biopsy. Weakly supervised end-to-end methods have made enormous progress in the last 5 years and have been used successfully in numerous tumor entities (21, 24, 25). Today, the first commercial products using such methods are already on the market, including a DL system for predicting clinical outcome and genetic alterations such as microsatellite instability status from colorectal cancer H&E slides (26, 27).

In contrast to previous studies, we developed a weakly supervised DL-based quantification of PD-L1 status in IHC images from patients with non–small cell lung cancer (NSCLC) and evaluated it in a cohort of patients with solid tumors, all of them treated with immunotherapy. Thereafter, we investigated the performance of the DL-based PD-L1 scoring to stratify patient response to immunotherapy.

## Materials and Methods

### Ethics Statement

This study was performed in accordance with the Declaration of Helsinki. The overall analysis was approved by the Ethics commission of the Medical Faculty of Technical University of Dresden (BO-EK-444102022). The Vall d'Hebron Institute of Oncology (VHIO) Institutional Review Board approved this study for retrospective data [PR(AG)70/2018] and the prospective PREDICT study [PR(AG)29/2020]. All patients included in the clinical trials provided written informed consent. Need for informed consent for the computational analysis of the images in the retrospective study was waived.

### Cohort Description

We included PD-L1–stained IHC digitalized slides of patients with NSCLC from the publicly available NSCLC-Memorial Sloan Kettering (MSK) MIND cohort (28) with PD-L1 tumor proportion score (TPS) assessed by a pathologist. All patients were treated with immune checkpoint inhibitors (ICI) in monotherapy or in combination with other immunotherapies. Progression-free survival (PFS) was assessed as the time between therapy starting date and the progression date.

**FIGURE 1:**
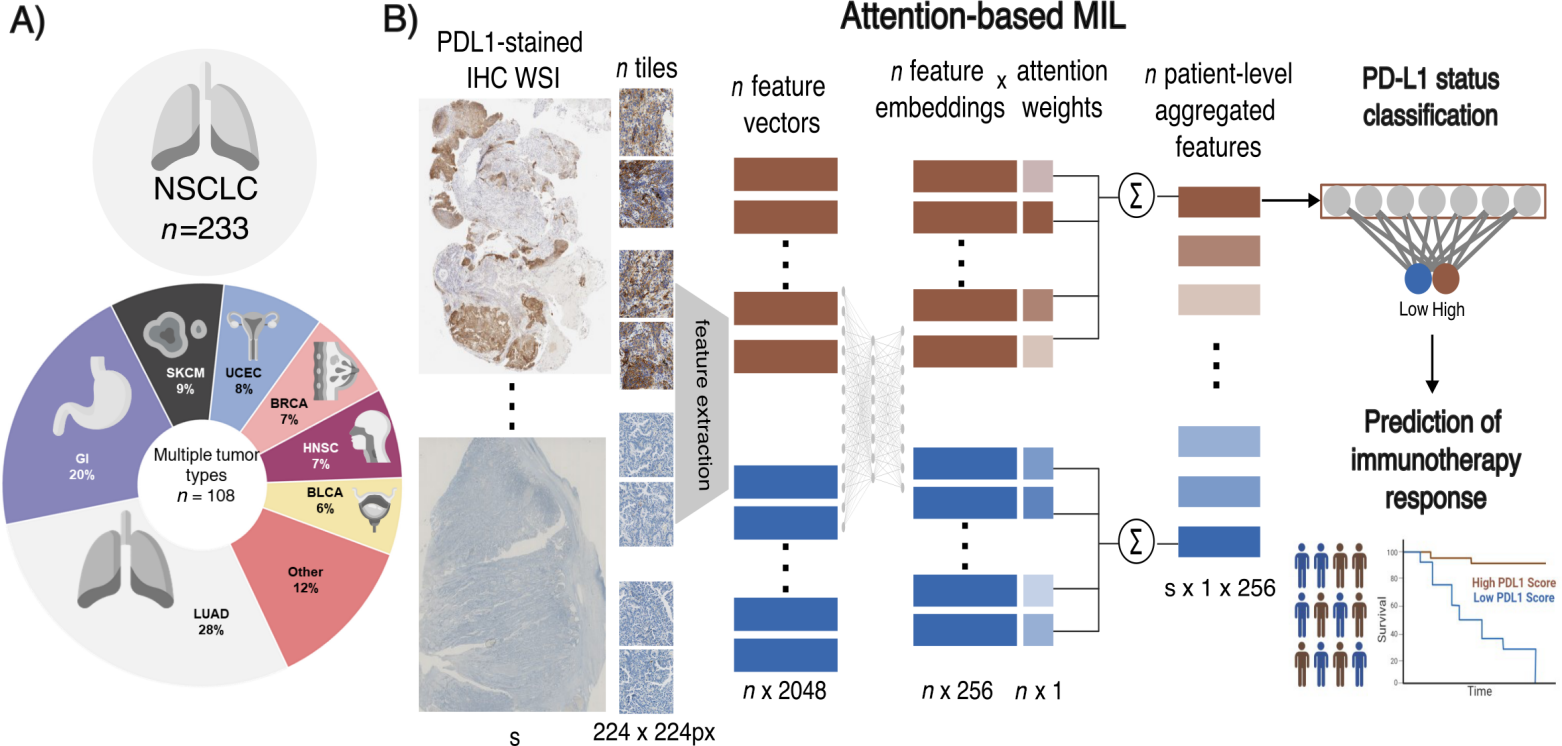
Overview of the study design. **A,** Population description of the NSCLC-MSK (training) and pan-cancer-VHIO (test) cohorts. **B,** Workflow of the attention-based MIL pipeline to classify PD-L1 status on IHC slides. The predicted PD-L1 status was investigated as predictive biomarker of response to immunotherapy.

As an external validation set, we collected data from patients with different tumor types (including NSCLC, gastrointestinal cancer, and melanoma among others) treated with ICIs at VHIO between 2012 and 2021. Only patients with tissue available from the biopsy prior to treatment and with PD-L1 TPS assessed by a pathologist were included in the analysis. In the external cohort, the combined positive score (CPS) was also collected when available. Biopsies at multiple time points during ICIs treatment from the same patient were collected and included in the PD-L1 status prediction performance. For those patients with multiple biopsies, only the samples closest to the start of treatment with ICIs were considered for treatment response prediction. An overview of the included population is described in Fig. 1A.

### PD-L1 IHC

In the NSCLC-MSK cohort, PD-L1 IHC staining of the slides was performed as described by Vanguri and colleagues (28). Briefly, the IHC was performed using the Bond III, Leica automated immunostaining platform using the PD-L1 antibody (E1L3N; Cell Signaling Technology). In the pan-cancer-VHIO cohort, 3.5-µm-thick slides stained with PD-L1 IHC were obtained from formalin-fixed paraffin-embedded tumor blocks of the VHIO cohort. The staining was performed with PD-L1 (SP263) Rabbit Monoclonal Primary Antibody (#790-4905, VENTANA) on the Benchmark ULTRA automated immunostaining platform. The OptiView DAB IHC Detection Kit (#760-700; VENTANA) was used for detecting the primary antibody. Placental and tonsil tissues were used as positive controls for all the stainings. The slides were digitalized using NanoZoomer 2.0-HT slide scanner by Hamamatsu Photonics, with a magnification of 20 × obtaining which corresponds to a pixel size of 0.46 µm × 0.46 µm. A trained pathologist (S. Mauchanski) interpreted the slides and provided the TPS and CPS for each sample. A negative score was defined as staining in less than 1% of tumor cells or the absence of staining in tumor cells. Slides with less than 100 tumor cells were excluded from PD-L1 CPS/TPS assessment. Investigators were blinded to patient response during the PD-L1 assessment.

### Image Preprocessing

In the NSCLC-MSK cohort, regions of interest (ROI) of the patient sample tissue were provided from the HALO software (Indica Labs). In the VHIO cohort, samples were annotated in polygonal ROIs using the open source QuPath software v0.3.2. These annotations were used to crop the image excluding any other tissue from the WSI that was not from the patient, that is, reference samples. For both cohorts, PD-L1–stained WSIs were tessellated into nonoverlapping tiles of 224 × 224 pixels corresponding to an edge length of 256 µm from the ROIs. Blurry tiles or tiles without tissue samples, defined as tiles containing less than 2% of edges in the image, were discarded using Canny edge detection (29–33).

### PD-L1 Status Classification Model

We developed weakly supervised DL methods to classify high (TPS ≥ 1%) and low (TPS < 1%) PD-L1 status from PD-L1–stained IHC samples. The DL approach consisted of two steps: first, feature vectors were extracted from every tile of the WSI, obtaining a tile-level feature vector. Thereafter, we performed weakly supervised feature aggregation and classification.

Feature extraction was performed using the Retrieval with Clustering-guided Contrastive Learning (RetCCL) model (34), an ImageNet-weighted ResNet50 pretrained in a self-supervised manner on a pathology dataset with 32,000 H&E-stained histology slides across various cancer types. After feature extraction, vectors of 2,048 features were obtained from every 224 × 224 pixels tile image. The attention-based multiple instance learning (MIL) model (35) consisted of an attention-based mechanism, followed by a classification head. First, a trainable weight is assigned to each tile-level feature vector, referred to as attention. Then, the features and weights were aggregated by the attention scores of the tile-level features to obtain the attention-weighted embeddings per sample. Finally, the embeddings were given to the classification head to obtain the model's sample-level prediction scores.

The model was trained using 5-fold cross-validation, with each fold stratified to maintain a proportional representation of PD-L1 status in the training, validation and test set. The area under the ROC (AUC) metric was used to evaluate the models. In the external validation cohort, we ran inference from the five models from cross-validation and the prediction scores were averaged to predict the PD-L1 status. For explainability analysis, the model with better performance on the training set was selected. We investigated the performance of the model when it is trained in the pan-cancer-VHIO cohort and validated in the NSCLC-MSK cohort following the same methods.

### Response Prediction Analysis and Explainability

The association between PFS and PD-L1 status was investigated using Cox proportional-hazards model and Kaplan–Meier curves with DeLong method in both the NSCLC-MSK and the pan-cancer-VHIO cohort. For explainability and visualization purposes, we computed high-resolution attention heat maps on the WSI as reported previously (29). Attention heat maps highlight the areas of the WSI where the model paid more attention for the decision-making. To compute high-resolution heat maps, the attention MIL model architecture was loaded into a fully convolutional equivalent with the trained weights. The equivalent convolutional neural network with attention MIL weights is running inference with a kernel size of 32 × 32 across the WSI. This approach allows for higher resolution attention heat maps than using the original tiles of 224 × 224 pixels used to train the model for visualization purposes. We compared the attention heat maps with PanCytokeratin-stained slides that highlight tumor areas. In addition, to assess whether the diversity of tumor types in the external cohort was affecting the performance of the model in predicting PD-L1, we studied the ratio of true and false positive and true and false negative of the model across tumor types. To have a better understanding of the model performance, an expert pathologist manually inspected the attention heat maps to find which morphologic factors the model is identifying to make predictions, including cases from false positive and false negative where the model finds confounding factors in the images. Finally, the predicted scores were compared as continuous variables with both TPS and CPS. Figure 1B shows the workflow of the methods.

### Data Availability Statement

All data from NSCLC-MSK cohort are publicly available at https://www.synapse.org/#!Synapse:syn26642505. All other images are available from the respective study principal investigators upon reasonable request. All source code for image preprocessing is publicly available at https://github.com/KatherLab. The tesselation script is available at https://github.com/KatherLab/preprocessing-ng. Extraction of RetCCL features was carried out with scripts from https://github.com/KatherLab/marugoto as well as the weakly supervised DL model and the heat maps for explainability used for the study.

## Results

### Cohort Clinical Data Description

We included 233 patients from the NSCLC-MSK cohort treated with ICIs with available PD-L1–stained IHC slides and TPS assessed by a pathologist. One IHC slide was discarded because of scarce tissue sample for image analysis. The median PFS was 3.2 [95% confidence interval (CI): 2.6–4.7] months with 30% (70/233) of patients responding to ICIs (i.e., achieved complete or partial response by RECIST 1.1). To test the PD-L1 status classifier in an external independent cohort, 122 PD-L1–stained slides from 108 patients from the pan-cancer-VHIO cohort were collected. We selected baseline samples from 106 patients treated with ICIs in monotherapy or in combination with other ICIs that had evaluable response to predict response to immunotherapy. The pan-cancer-VHIO cohort had a median PFS of 2.73 (95% CI: 1.84–3.98) months, with 15% (16/106) of patients with partial or complete response to ICIs. The cohort description is reported in Table 1.

**TABLE 1:** Clinical population characteristics for NSCLC-MSK and pan-cancer-VHIO cohort

Cohort	NSCLC-MSK	pan-cancer-VHIO
*N*	233	108 (106 treated with immunotherapy)
Age, median (range)	68 (30–93)	57 (29–77)
Sex		
Male	102	57
Female	131	51
Treatment		
Anti-PDL1	N/A	35
Anti-PD1	N/A	13
Anti-PD1 + other ICIs	N/A	30
Anti-PDL1 + other ICIs	N/A	8
Other ICIs	N/A	14
Anti-PD1 + chemotherapy	N/A	4
Other combinations	N/A	4
Tumor type		
Lung	233	27
Gastrointestinal (including gastric, colon, pancreas)		22
Melanoma		10
Gynecologic (including ovarian, uterine, and endometrial)		9
Breast		8
Urinary		6
Head and neck		8
Hepatobiliary		4
Sarcoma		3
Other		9
Biopsy location		
Primary	105	69
Metastatic	128	39
PD-L1 expression		
TPS < 1%	73	59
TPS ≥ 1%	163	49
Best response		
CR/PR	70	16
SD/PD	154	90
PFS in months [median (95% CI)]	3.2 (2.6–4.7)	2.73 (1.84–3.98)

Abbreviations: CI, confidence interval; CR, complete response; ICI, immune checkpoint inhibitors; N/A, not available; PD, progressive disease; PR, partial response; SD, stable disease; TPS, tumor proportion score.

### Weakly Supervised DL Approach to Classify PD-L1 Status Predicts Response to ICIs

**FIGURE 2:**
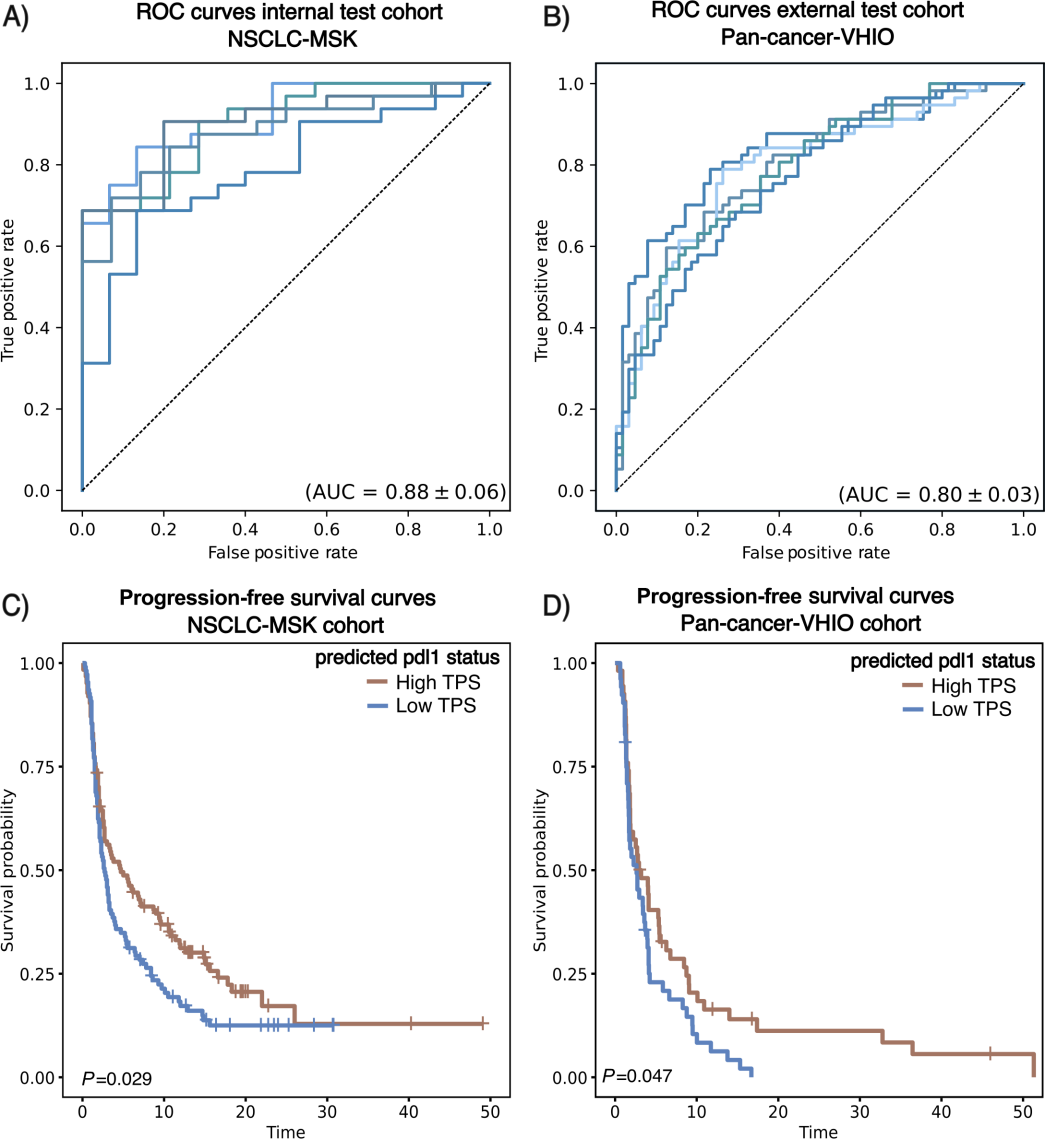
Performance overview of the model for predicting PD-L1 status and response to immunotherapy in NSCLC-MSK and pan-cancer-VHIO cohort. AUC curves for the model to predict PD-L1 status (TPS ≥ 1%) in the training (NSCLC-MSK cohort; **A**) and in the test cohort (pan-cancer-VHIO cohort; **B**) for the 5-folds cross-validation. All trained models were deployed in the test cohort. Kaplan–Meier curves for the predicted PD-L1 status (high/low) from the model differentiates patients with longer PFS to immunotherapy from patients with shorter survival in both NSCLC-MSK (**C**) and pan-cancer-VHIO cohort (**D**).

Our attention MIL-based model was trained on PD-L1–stained IHC images from the NSCLC-MSK cohort to predict PD-L1 status TPS>1%, reaching a high performance with a cross-validated mean AUC of 0.88 ± 0.06 (Fig. 2A). Subsequently, we explored how the model generalizes in an external cohort (pan-cancer-VHIO), which consisted of different tumor types, including NSCLC. We deployed all the models from cross-validation in the IHC images of the external pan-cancer-VHIO achieving a mean AUC of 0.80 ± 0.03 (Fig. 2B). The detailed performance statistics are available in Supplementary Table S1 (accuracy, sensitivity, specificity, positive predictive value, negative predicted value). Overall, these results show that the PD-L1 classifier trained on the NSCLC can generalize to broader and more diverse datasets, even when stained with different protocols. Similar results were obtained when training on the pan-cancer-VHIO cohort and validating on the NSCLC-MSK (Supplementary Fig. S1).

We investigated whether the predicted PD-L1 status associated with response to immunotherapy in terms of PFS endpoint. A strong association between PD-L1 predicted scores and response to ICIs is observed in both NSCLC-MSK cohort [HR: 1.4 (95% CI: 1–1.8), *P* = 0.03] and pan-cancer-VHIO cohort [HR: 1.5 (95% CI: 1–2.3), *P* = 0.049], as measured by Cox regression analysis. In the external validation cohort, the predicted PD-L1 status showed more significant association with response to immunotherapy compared with TPS [HR: 1.4 (95% CI: 0.96–2.2), *P* = 0.082] and CPS [HR: 1.2 (95% CI: 0.79–1.9), *P* = 0.386]. In addition, patients classified as high PD-L1 status showed significant longer median PFS compared with patients classified as low PD-L1 status in both the training and test cohorts by Kaplan–Meier curves and long-rank test; median PFS of 4.70 [interquartile range (IQR): 2.7–9.0] versus 2.7 (IQR: 2.1–3.5) months, *P* < 0.05 and 3.06 (IQR: 1.84–5.46) versus 2.63 (IQR: 1.71–3.94) months, *P* < 0.05; respectively (Fig. 2C and D). In summary, these results suggest that the predicted PD-L1 status can discriminate which patients are more likely to benefit from ICI treatment.

### Explainability Analysis: PD-L1 Predicted Status Differentiates Tumor Cells from Surrounding Tissue

**FIGURE 3:**
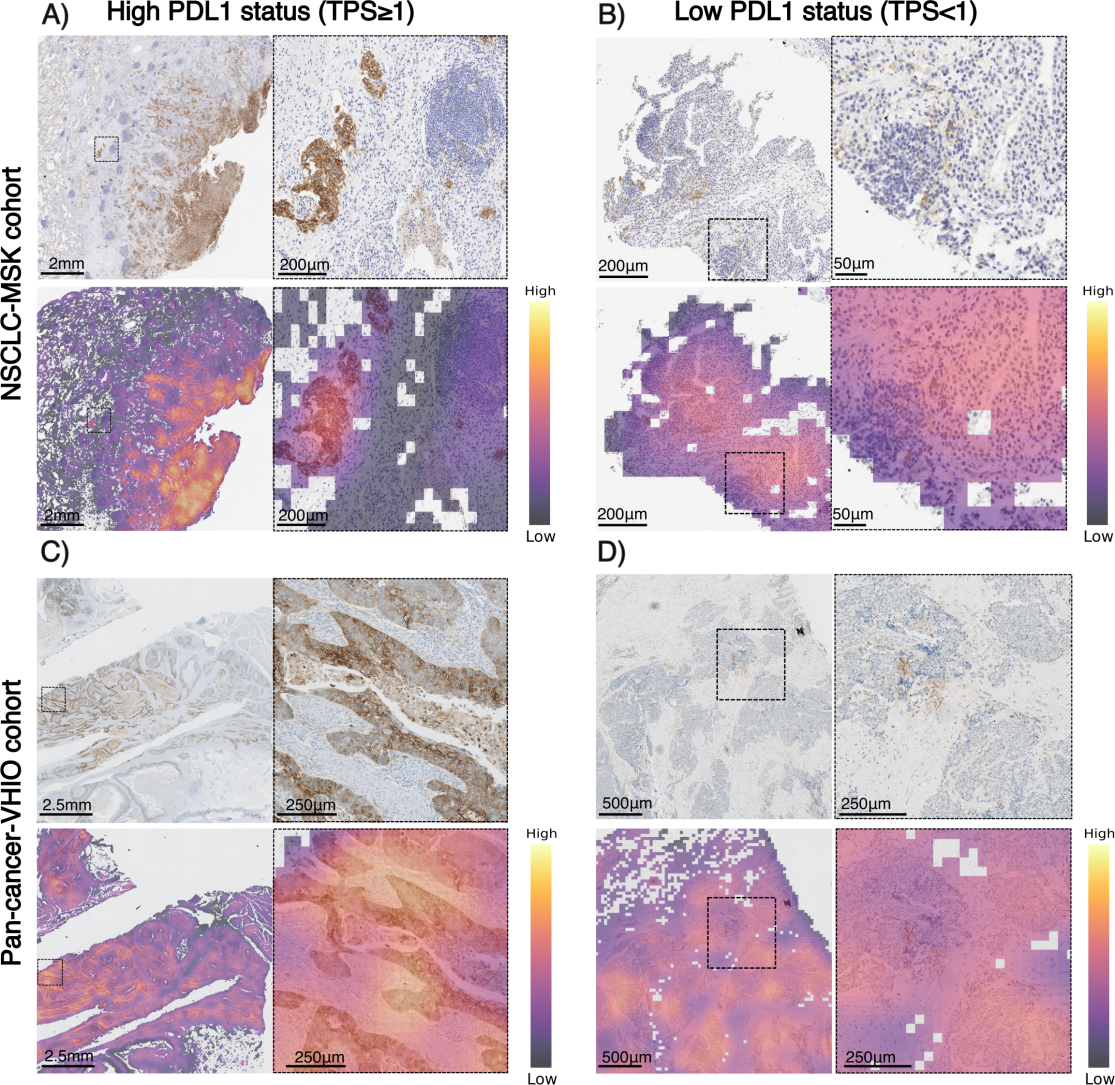
Visualization of high and low PD-L1 score patients from the NSCLC-MSK and pan-cancer-VHIO cohort. Magnification of tumor areas with the highest attention scores for both high PD-L1 (TPS ≥ 1%) and low PD-L1 (TPS < 1%) samples from the training and validation cohort. Magnification shows that for both high and low scores the model gives more attention to tumor cells, ignoring areas of high lymphocyte density [**A**: tumor cells (high attention, left), lymphocytes (low attention, right); **B** and **D**: tumor cells (high attention, right), lymphocytes (low attention, left); **C**: tumor cells (high attention), stroma (low attention)] (Image magnification, **A**: 1.25×–6.12×, **B**:, **C**: 1.25×–2.5×, **D**: 2.5×–5.0×).

The assessment of PD-L1 status can be performed considering the PD-L1 expression only from the tumor cells (TPS) or including both tumor cells and surrounding tissue, such as the stroma component (CPS). The classifier was trained on a NSCLC cohort where PD-L1 was assessed only from tumor cells (TPS) as standard of care. Then, the model was validated in a pan-cancer cohort, in which PD-L1 status was assessed with both TPS and CPS. For explainability analysis of the classifier, we investigated the distribution of attention scores in some of the patients that were correctly classified as high and low PD-L1 status in both NSCLC-MSK and pan-cancer-VHIO cohort. A pathologist review identified the model's capability to correctly identify tumor cells with and without expression of PD-L1 in the membrane and that it can differentiate tumor cells expressing PD-L1 from other cellular structures such as the stromal tissue, adipose tissue, or immune cells in the tumor microenvironment (Fig. 3). This visual analysis was further validated through a comparative examination of attention heat maps and PanCytokeratin-stained images (Supplementary Fig. S2). Taken together, these results suggest that even when the PD-L1 is expressed in the immune component, the model will recognize that tissue as nontumoral and will not consider it as relevant for the final classification, which means that our DL model is more sensitive to TPS than CPS.

**FIGURE 4:**
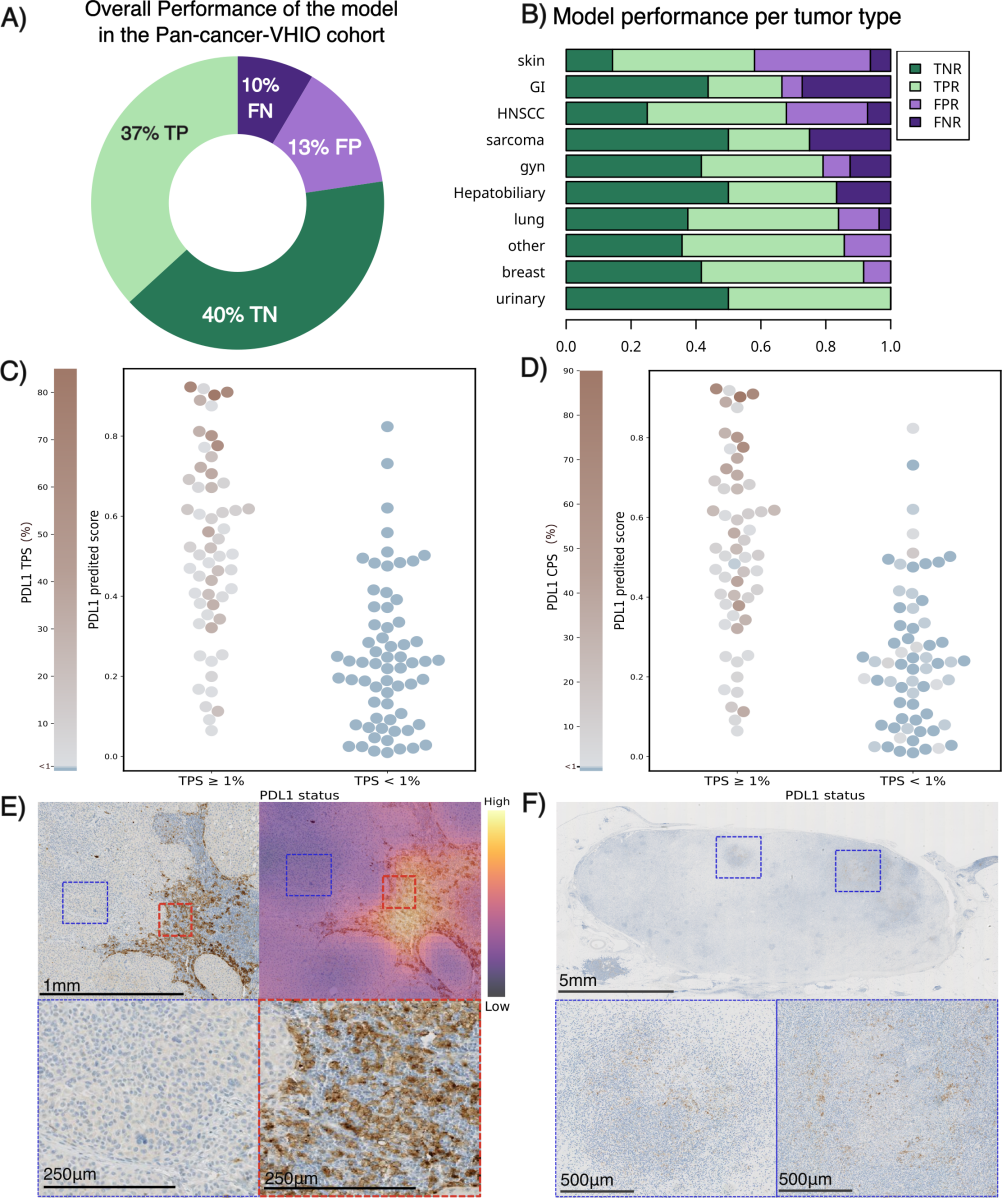
Explainability for model performance in the pan-cancer-VHIO validation cohort. Overall performance of the model in the validation cohort (**A**). Distribution of true negative ratio (TNR), true positive ratio (TPR), false negative ratio (FNR), and false positive ratio (FPR) per tumor type in the pan-cancer-VHIO validation cohort (**B**). Distribution of predicted scores in the pan-cancer-VHIO cohort compared with continuous TPS (**C**) and CPS (**D**). Examples of false positives due to histologic differences from NSCLC: PD-L1–stained IHC image from melanoma tumor sample predicted as high PD-L1 by the model (predicted TPS ≥ 1%) with low PD-L1 expression in the tumor cells (TPS < 1%) and high PD-L1 expression in the immune component (CPS ≥ 1%). Magnification of areas with tumor cells with no PD-L1 expression (blue) and immune cells with PD-L1 expression (red), image magnification: 5× and 20× (**E**). Examples of false negative due to low cellularity samples: PD-L1–stained IHC image from gastrointestinal tumor sample predicted as low PD-L1 by the model (predicted TPS < 1%) with high PD-L1 expression in the tumor cells (TPS = 20%) and high PD-L1 expression in the immune component (CPS ≥ 35%). Magnification of regions with tumor cells, image magnification, 0.35× and 5× (**F**).

Furthermore, the model showed an overall accuracy of 77% to correctly classify samples with high and low PD-L1 expression in the tumor cells (Fig. 4A; Supplementary Table S1). However, as shown in Fig. 4, when testing the model in a heterogeneous cohort such as the pan-cancer-VHIO dataset, some potential factors may limit the overall performance of the model. As can be seen in Fig. 4B and Supplementary Table S1, the model's classification performance is weaker in tumor histologies such as gastrointestinal tumor, melanoma, and sarcoma. These results are likely to be related to the histologic and molecular differences between these tumor types and the NSCLC, which is the tumor type used to train the model.

Samples with inconsistent PD-L1 expression between the tumor cells and the stromal tissue (e.g., high CPS and low TPS) also pose a challenge to the model. In some of these cases, a pathologist review identified that the DL model recognizes the PD-L1 expression from the stromal component as high PD-L1 status (Fig. 4C and D). These findings may also be linked to histologic differences in population. Higher false positive rates correspond to differentiated tumor types such as melanoma, head and neck, and other tumor types. Figure 4E shows magnification of a PD-L1–stained IHC sample from a melanoma biopsy. In this case, the model fails to recognize the tumor cells from skin (blue magnification) and sees the immune dense component (red magnification) as tumor area, classifying the patient as high PD-L1 when the ground truth based on TPS is low PD-L1. Other misclassification cases correspond to samples with TPS falling near the classification threshold (TPS < 1%) as shown in Fig. 4C (77% of the false negative cases had a low value of TPS, ranging between 1% and 7%) or samples with low cellularity as shown in Fig. 4F, where the model fails to classify as low due to the large amount of sample without PD-L1 expression. Together, these results indicate that the trained model on the NSCLC-MSK cohort generalizes to a more diverse cohort and pays attention to relevant tissue components, that is, focused on tumor cells.

## Discussion

IHC analysis of PD-L1 expression in tumor samples is the most commonly used biomarker in clinical practice and currently employed as patient selection for some of the standard ICI therapies (11, 36). However, the manual evaluation of this biomarker results in interobserver variability, which may lead to patient misclassification in terms of treatment options (7). Therefore, there is a need for quantitative and standardized tools to evaluate PD-L1 status in clinical practice.

In this study, we implemented and validated a weakly supervised DL method that can predict PD-L1 status in patients with several tumor types. The current study found that a DL model trained with weak labels for predicting TPS in a NSCLC cohort is capable of differentiating between tumor cells and immune cells in most of the cases and correctly quantifying the expression of PD-L1 in the specific tumor cells. This is a particularly encouraging result that supports the idea of using weak labels to classify WSI without the need of delineating structures, as done in previous studies (14, 15). Interestingly, the model proves generalization to other cohorts across different tumor types, not limited to lung, and across two different PD-L1 antibodies. Furthermore, we proved that the predicted PD-L1 status from our model can help to identify patients that would benefit from immunotherapy.

Despite the promising findings of our study, several limitations should be acknowledged. First, the model was trained and tested in a relatively small cohort, which may limit the accuracy and generalizability of the model. In addition, the evaluation of the model in different histologic tumor types might influence the performance. The model showed lower performance in the tumor types such as melanoma, gastrointestinal, sarcomas, head and neck, which may be related to the different biology and histology of these tumor types, compared with NSCLC (37, 38). Moreover, differences in staining protocols between laboratories and tumor types might contribute to the higher rate of misclassifications when evaluating the model (10, 38). For example, the presence of nonspecific staining such as melanin in the case of melanoma, tumor necrosis, or dense lymphoid aggregates could be potential sources of biases for the model. Finally, the features used in the DL model were trained using H&E images and applied to IHC images. Even though the model shows a good performance using RetCCL features, this could be limiting the model to achieve enhanced classification. Nonetheless, despite all the limitations encountered in the heterogeneous external cohort, our study demonstrates that a weakly supervised model, trained on NSCLC, can accurately classify PD-L1 status in most cases.

Furthermore, the differences in standard methods for assessing PD-L1 based on tumor type and treatment regime could influence the performance of DL model as an immunotherapy response biomarker when applied to a diverse cohort of tumor types. Although the model was originally developed in a NSCLC cohort, where PD-L1 status is a gold standard, a widely accepted biomarker used in the clinical routine for treatment decision, it was tested on a pan-cancer cohort with advanced patients from clinical trials. In this setting, the expression of PD-L1 in the tumor cells (TPS) as a response biomarker remains unclear, and it is often evaluated using CPS, which included both stromal and tumoral PD-L1 expression. The use of CPS in more advanced patients allows for broader inclusion of patients into clinical trials (39, 40). Interestingly, our model showed acceptable performance for predicting response to immunotherapy in both NSCLC and pan-cancer cohort.

Nevertheless, to address the previous constraints related to cohort characteristics, we investigated the performance of the model when trained on the pan-cancer-VHIO cohort and validated on the MSK-NSCLC cohort. However, the limitations of the pan-cancer-VHIO cohort, characterized by a small sample size, substantial tumor type heterogeneity, and the inclusion of predominantly advanced tumors accessible only via biopsy, may potentially reduce the reliability of the model performance.

One additional limitation we acknowledge is the potential impact of interobserver variability in model development and its bias toward subjective assessment. It is worth noting, however, that the PD-L1 measurements were sourced from clinical reports, which were assessed by multiple pathologists in routine clinical practice. This property enables our model to get insights from a wide range of pathologist evaluations, thereby mitigating bias from a single evaluation and accounting for interobserver variability. In terms of potential future work, these models could benefit from the integration of uncertainty modeling methods that consider the inherent variability of the ground truth during modeling.

To conclude, our findings differ from the previous studies mainly in terms of outcome prediction and methods implementation. Several studies have reported promising results and similar variability between the AI tool and the pathologist performance for assessing PD-L1 status (15). However, most of these methods require either manual or automatic annotation of the tumor tissue, as well as the detection and classification of the different cells in the sample. Despite other studies trying to address this limitations by proposing tissue classification as an annotation solution instead of using cell detection, our proposed method predicts PD-L1 status directly from the WSI, without any physician input or need for further stainings such as pan-cytokeratins. This one-step approach reduces the risk of errors, while yielding similar results to previously developed methods and providing explainability of the model by terms of attention. In addition, as a distinguishing characteristic of this study, it investigates the potential value of the DL tool to predict response to immunotherapy, which is the main objective of PD-L1 quantification.

This study supports the idea that weakly supervised DL methods are able to distinguish between the different histologic patterns in IHC and learn how to quantify expression of PD-L1 in tumor samples without any additional input from the physician. However, further research is needed in larger and more diverse cohorts for the model to gain a better understanding of the different tumor histologies, so it can properly generalize to any tumor type.

## Supplementary Material

Table S1 Model classification performance for the training (NSCLC-MSK) and test (pan-cancer-VHIO) cohorts: Accuracy, Sensitivity, Specificity, Positive Predictive Value (PPV) and Negative Predictive Value (NPV). For the Pan-cancer cohort, the performance for the different tumor types is also reported.

Figure S1 Performance overview of the model for predicting PD-L1 status and response to immunotherapy when trained on pan-cancer-VHIO cohort and validated in NSCLC-MSK. Area under the receiver operating characteristic (ROC) curves for the model to predict PD-L1 status (TPS≥1%) in the training (pan-cancer-VHIO cohort) (A) and in the test cohort (NSCLC-MSK cohort) (B) for the 5-folds cross-validation. All trained models were deployed in the test cohort. Kaplan-Meier curves for the predicted PD-L1 status (high/low) differentiates patients with longer Progression Free Survival to immunotherapy from patients with shorter survival in both pan-cancer-VHIO (C) and NSCLC-MSK cohort (D).

Figure S2 Visualization of PanCytokeratin IHC staining, PD-L1 IHC staining and model attention heatmaps of a high (top) and low (bottom) PD-L1 score patients pan-cancer-VHIO cohort. PanCytokeratin IHC stained images differentiate tumor tissue (A, D). PD-L1 IHC stained images highlight tumor cells with high PD-L1 expression (B, E). Attention heatmaps indicate that the model is considering tumor areas for evaluating TPS (C,F)
