# Supplementary material for: Weakly Supervised Deep Learning Predicts Immunotherapy Response in Solid Tumors Based on PD-L1 Expression
Source: Cancer Res Commun. 2024 Jan 11;4(1):92–102. doi: 10.1158/2767-9764.CRC-23-0287 (PMC10782919; doi:10.1158/2767-9764.CRC-23-0287)
Supplement: Table S1 — Model classification performance for the training (NSCLC-MSK) and test (pan-cancer-VHIO) cohorts: Accuracy, Sensitivity, Specificity, Positive Predictive Value (PPV) and Negative Predictive Value (NPV). For the Pan-cancer cohort, the performance for the different tumor types is also reported. [file crc-23-0287-s01.pdf]

| | N | ACCURACY | SENSITIVITY | SPECIFICITY | PPV | NPV |
|---|---|---|---|---|---|---|
| **NSCLC-MSK** | 233 | 0.75 | 0.71 | 0.85 | 0.91 | 0.57 |
| **PANCANCER-VHIO** | 122 | 0.77 | 0.79 | 0.75 | 0.74 | 0.80 |
| **LUNG** | 30 | 0.83 | 0.93 | 0.73 | 0.76 | 0.92 |
| **GASTROINTESTINAL** | 27 | 0.68 | 0.44 | 0.85 | 0.67 | 0.69 |
| **SKIN** | 15 | 0.60 | 1.00 | 0.33 | 0.50 | 1.00 |
| **GYNECOLOGICAL** | 10 | 0.78 | 0.67 | 0.83 | 0.67 | 0.83 |
| **HEAD AND NECK** | 9 | 0.88 | 1.00 | 0.50 | 0.86 | 1.00 |
| **BREAST** | 8 | 0.88 | 1.00 | 0.83 | 0.67 | 1.00 |
| **URINARY** | 6 | 1.00 | 1.00 | 1.00 | 1.00 | 1.00 |
| **HEPATOBILIARY** | 5 | 0.75 | 0.50 | 1.00 | 1.00 | 0.67 |
| **SARCOMA** | 3 | 0.67 | 0.50 | 1.00 | 1.00 | 0.50 |
| **OTHER** | 9 | 0.71 | 1.00 | 0.60 | 0.50 | 1.00 |

**Table S1.** Model classification performance for the training (NSCLC-MSK) and test (pan-cancer-VHIO) cohorts: Accuracy, Sensitivity, Specificity, Positive Predictive Value (PPV) and Negative Predictive Value (NPV). For the Pan-cancer cohort, the performance for the different tumor types is also reported.
