# Supplementary material for: Weakly Supervised Deep Learning Predicts Immunotherapy Response in Solid Tumors Based on PD-L1 Expression
Source: Cancer Res Commun. 2024 Jan 11;4(1):92–102. doi: 10.1158/2767-9764.CRC-23-0287 (PMC10782919; doi:10.1158/2767-9764.CRC-23-0287)
Supplement: Figure S1 — Performance overview of the model for predicting PD-L1 status and response to immunotherapy when trained on pan-cancer-VHIO cohort and validated in NSCLC-MSK. Area under the receiver operating characteristic (ROC) curves for the model to predict PD-L1 status (TPS≥1%) in the training (pan-cancer-VHIO cohort) (A) and in the test cohort (NSCLC-MSK cohort) (B) for the 5-folds cross-validation. All trained models were deployed in the test cohort. Kaplan-Meier curves for the predicted PD-L1 status (high/low) differentiates patients with longer Progression Free Survival to immunotherapy from patients with shorter survival in both pan-cancer-VHIO (C) and NSCLC-MSK cohort (D). [file crc-23-0287-s02.pdf]

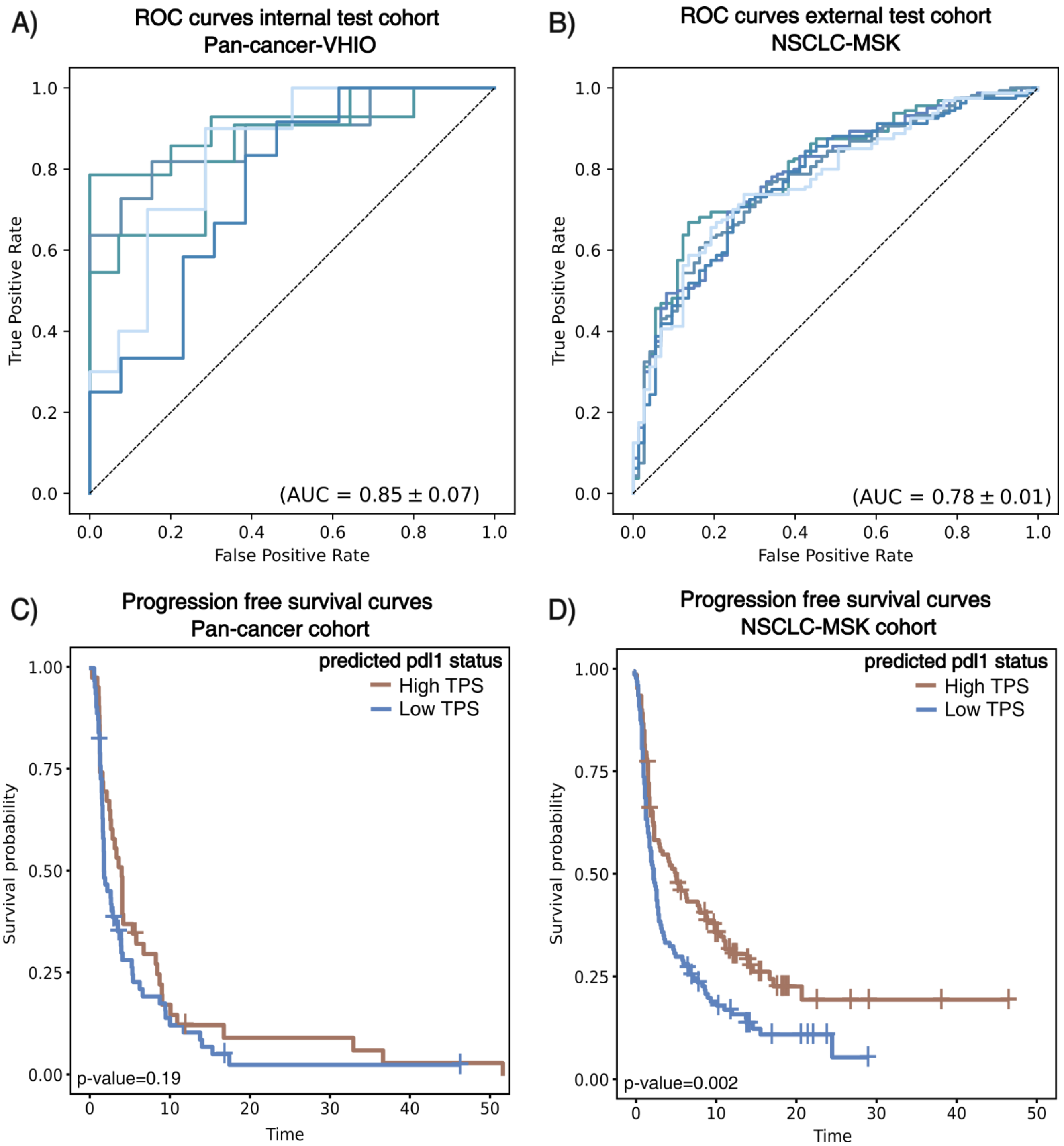

Figure S1. Performance overview of the model for predicting PD-L1 status and response to immunotherapy when trained on pan-cancer-VHIO cohort and validated in NSCLC-MSK. Area under the receiver operating characteristic (ROC) curves for the model to predict PD-L1 status (TPS≥1%) in the training (pan-cancer-VHIO cohort) (A) and in the test cohort (NSCLC-MSK cohort) (B) for the 5-folds cross-validation. All trained models were deployed in the test cohort. Kaplan-Meier curves for the predicted PD-L1 status (high/low) differentiates patients with longer Progression Free Survival to immunotherapy from patients with shorter survival in both pan-cancer-VHIO (C) and NSCLC-MSK cohort (D).
