# Supplementary material for: Weakly Supervised Deep Learning Predicts Immunotherapy Response in Solid Tumors Based on PD-L1 Expression
Source: Cancer Res Commun. 2024 Jan 11;4(1):92–102. doi: 10.1158/2767-9764.CRC-23-0287 (PMC10782919; doi:10.1158/2767-9764.CRC-23-0287)
Supplement: Figure S2 — Visualization of PanCytokeratin IHC staining, PD-L1 IHC staining and model attention heatmaps of a high (top) and low (bottom) PD-L1 score patients pan-cancer-VHIO cohort. PanCytokeratin IHC stained images differentiate tumor tissue (A, D). PD-L1 IHC stained images highlight tumor cells with high PD-L1 expression (B, E). Attention heatmaps indicate that the model is considering tumor areas for evaluating TPS (C,F) [file crc-23-0287-s03.pdf]

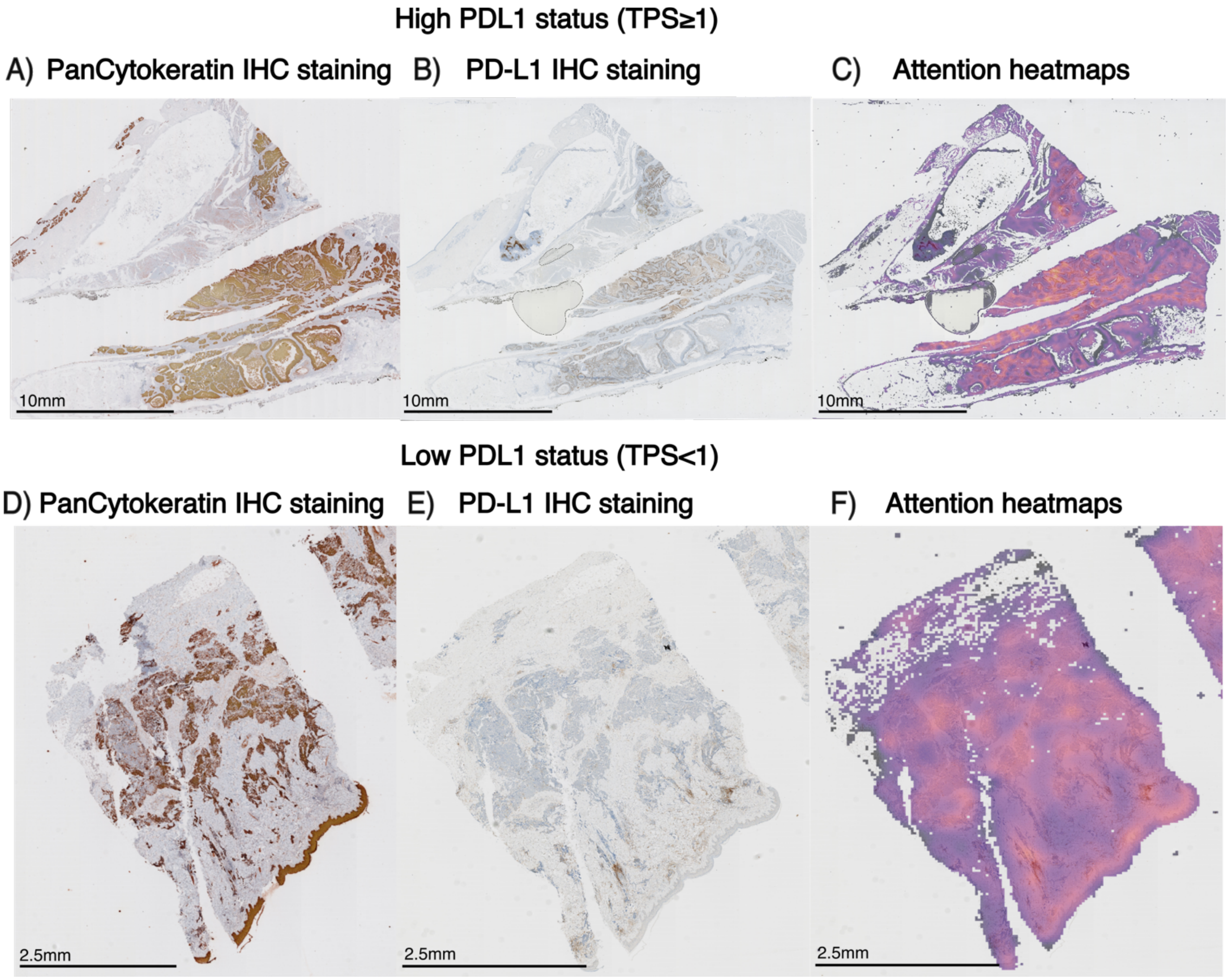

Figure S2. Visualization of PanCytokeratin IHC staining, PD-L1 IHC staining and model attention heatmaps of a high (top) and low (bottom) PD-L1 score patients pan-cancer-VHIO cohort. PanCytokeratin IHC stained images differentiate tumor tissue (A, D). PD-L1 IHC stained images highlight tumor cells with high PD-L1 expression (B, E). Attention heatmaps indicate that the model is considering tumor areas for evaluating TPS (C,F)
